# Preparation of Specialty Malt Through Explosive Puffing: Characterization of Quality Attributes and Its Effect on Beer Quality

**DOI:** 10.3390/foods15061113

**Published:** 2026-03-23

**Authors:** Qi Pan, Jiangyu Zhu, Yongqi Yin, Zhengfei Yang

**Affiliations:** School of Food Science and Engineering, Yangzhou University, Yangzhou 225000, China

**Keywords:** grain processing, beer, cereal processing, brewing

## Abstract

Driven by the growing demand for flavor diversification in the global craft beer market, conventional drum roasting for specialty malt faces limitations in time consumption and flavor retention. This study aimed to explore explosive puffing as a novel approach for specialty malt production. Base barley malt was treated via explosive puffing at 0.8 MPa to prepare puffed specialty malt, followed by comprehensive characterization of its physicochemical properties, volatile profile, and antioxidant activity, with brewing trials conducted at 15% grist substitution. Results showed that puffed malt reached a color of 183.15 EBC, with formation of roasted pyrazines and caramel-like furans, and a nearly 3-fold increase in total phenolic content and antioxidant capacity. At 15% addition, the puffed malt maintained wort free amino nitrogen and reducing sugar levels, while significantly enhancing beer color, roasted aroma, and antioxidant activity. These findings demonstrate that explosive puffing is a promising alternative to conventional roasting for producing specialty malt.

## 1. Introduction

Driven by the growing demand for diverse flavor profiles in the craft beer market, specialty malt has become a core ingredient for product differentiation. Persistence Market Research projects that the global specialty malts market will generate revenue of approximately USD 2.83 billion in 2025 [1]. With a compound annual growth rate of 5.3%, the market is forecast to reach a valuation of USD 4.06 billion by the end of 2032. Unlike base malt, which is produced under relatively mild conditions, specialty malt is subjected to high-temperature treatment during its production [2,3]. This intense heating promotes non-enzymatic browning reactions that generate a variety of substances responsible for specialty malt’s characteristic color and complex aroma [4,5,6]. Moreover, studies have shown that thermal treatment can enhance the antioxidant capacity of cereals by facilitating the release of bound phenolic compounds [7]. However, the high-temperature process also deactivates enzymes which limits the proportion of specialty malt that can be incorporated into the brewing grist [8]. Currently, the production of specialty malt primarily relies on roasting either green malt or finished base malt in dedicated roasting drums [9]. This conventional approach is hindered by its prolonged duration and the use of non-enclosed equipment, which inevitably causes extensive volatilization of aroma compounds. In our recent study, we attempted to adopt the microwave method for the preparation of specialty malt [10]. We confirmed that microwave treatment coupled with a mechanical tumbling step could enables rapid, energy-efficient production of specialty malts with enhanced color, antioxidant properties and roasted aroma. However, the application of microwave technology for specialty malt production is not without limitations. Its intermittent operation mode poses challenges for continuous industrial production, and the risk of local overheating remains a concern due to uneven energy distribution. These drawbacks prompted us to explore whether other thermal processing methods could serve as viable alternatives for specialty malt fabrication.

Explosive puffing is a technology employed globally in the snack food processing sector, with notable prevalence in East Asia. Unlike conventional roasting, which relies primarily on thermal conduction, explosive puffing applies simultaneous thermal and mechanical stress. In this process, food materials are heated inside a sealed rotating cylinder under elevated pressure. Once the target pressure is reached, the vessel is rapidly decompressed by opening the lid, resulting in the puffing of the product [11]. The synergy of high temperature and pressure during treatment trigger non-enzymatic browning reactions, which generate color and aroma compounds that enhance the sensory qualities of the product. Simultaneously, the process causes enzyme inactivation, starch gelatinization, and protein denaturation, while the formation of a porous structure enhances the release of internal bioactive components such as polyphenols [12]. Recent research has revealed that applying differential pressure explosive puffing to spent coffee grounds resulted in a significant enhancement of the craft beer’s flavor profile [13]. Another study has shown that explosive puffing is a viable alternative to conventional coffee bean roasting, resulting in a final product with enhanced antioxidant capacity [14]. In addition, steam explosion of barley bran has been shown to increase total phenolic content (TPC) [15]. Based on the distinctive features of explosive puffing, the combination of high temperature and pressure within a sealed vessel followed by rapid decompression, we hypothesized that this technology could offer advantages over conventional roasting for specialty malt production. We postulated that the shorter processing duration and closed processing system would enhance the retention of volatile compounds, and that the thermo-mechanical stress generated during rapid decompression would promote structural modifications, which may improve the extractability of bioactive compounds.

To evaluate the viability of explosive puffing for specialty malt production, base malt (BM) was processed with this technology. The processed malt underwent a set of analyses to determine its physicochemical parameters, antioxidant indicators, volatiles, and sensory attributes. A preliminary assessment of its brewing applicability was conducted.

## 2. Materials and Methods

### 2.1. Materials

BM (*Hordeum vulgare* L.) was sourced by Supertime-malting (Weifang, China). Hops (Tsingtao Flower and SA-1 pellets) were procured from Gansu Yasheng Lvxin Beer Ingredients Co., Ltd. (Jiuquan, China) and Xinjiang Sapporo Agricultural Science and Technology Development Company (Urumqi, China), respectively. The CN36 yeast was provided by Angel Yeast Co., Ltd. (Yichang, China).

### 2.2. Sample Development

#### 2.2.1. Development of Explosive Puffed Malt

**Figure 1:**
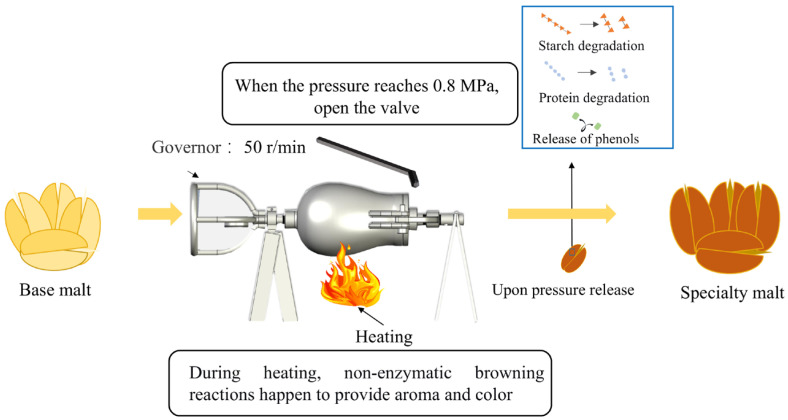
Illustration of explosive puffing of malt.

BM was processed using a cylindrical puffing apparatus (Baobaigu, BBG-520, Jinhua, China). 250 g of BM was loaded into the cylinder, which was rotated at 50 r min^−1^ and heated. The pressure was rapidly released once the internal pressure reached 0.8 MPa (8 min) (Figure 1). After cooling to ambient temperature, the processed samples were collected and stored in sealed polyethylene bags.

#### 2.2.2. Wort Preparation

Prior to mashing, malt was crushed using a roller mill (Hongborui brewing equipment, Hefei, China). Beers were brewed using either 100% BM or a grist with 15% explosive puffed malt (EM). 2.5 kg of the milled grain mass was mixed with 10 L of water at a 1:4 ratio and mashed according to a programmed mashing process. Mashing was initiated at 45 °C for 30 min, held at 63 °C for 60 min, and then raised to 72 °C for 30 min. The temperature was finally raised to 78 °C and held for 10 min before termination. The resulting wort was filtered and sparged with 2 L of hot water (78 °C). After filtration, the wort was boiled for 1 h. Hops were added at specific intervals: 3 g of Tsingtao Flower hops at the start of boiling and 3 g of SA-1 hops at 50 min. Post-boiling, the wort was adjusted to 12 °P and then cooled to 21 °C.

#### 2.2.3. Fermentation

Activated CN36 yeast was inoculated into 2 L aliquots of cooled wort at a dosage of 0.5 g per liter of wort. Fermentation was carried out in 3 L glass fermenters placed in a temperature-controlled incubator (Kegland, KL15813, Melbourne, Australia) at 21 °C for 7 days (primary fermentation). Following primary fermentation, the beers were bottled with the addition of 3 g sucrose per bottle and underwent a 10-d secondary fermentation at 21 °C, followed by storage at 4 °C.

### 2.3. Analytics

#### 2.3.1. Cereal Characterization

Physicochemical analysis of cereal samples was conducted in compliance with the standard protocols in Analytica-EBC [16]. Moisture content was determined by EBC 4.2. Color and extract were analyzed using EBC protocols: for BM, EBC 4.7.1 and EBC 4.5.1 were applied, respectively; for EM, EBC 5.6 and EBC 5.5 were used, respectively.

#### 2.3.2. Physicochemical Properties of Wort and Beer

Wort samples were analyzed for color, extract, and free amino nitrogen (FAN) content following EBC Methods 8.5, 8.3, and 8.10.1, respectively. The reducing sugar content was measured by the 3,5-dinitrosalicylic acid method [17].

Beer samples were characterized as follows: Alcohol, real degree of fermentation, color, and bitterness were determined according to EBC Methods 9.2.1, 9.5, 9.6, and 9.8, respectively. The pH was measured using a calibrated pH meter (Shanghai Yidian Scientific Instrument Co., Ltd., PHS-3C, Shanghai, China). Turbidity was assessed with a turbidimeter (Shanghai Xinrui Instrument Co., Ltd., WGZ-2PJ, Shanghai, China).

#### 2.3.3. Analysis of Volatile Compounds of Grain and Beer

Volatile profiles were characterized by headspace solid-phase microextraction coupled with gas chromatography-mass spectrometry (HS-SPME-GC-MS) (PerkinElmer, Clarus690-SQ8T, Waltham, MA, USA) employing a preconditioned (250 °C, 30 min) 75 μm CAR/PDMS fiber. This fiber was selected according to a previous study [18]. Grain (2 g) and beer (5 mL) samples were prepared in headspace vials with the addition of their respective internal standards: O-dichlorobenzene (5 μL of 130 mg/L) for grain and 3-methylbutan-1-ol (100 μL of 600 mg/L) for beer. For quantification, a relative semi-quantification method based on internal standard was adopted. Following a 10-min equilibration at respective temperatures (60 °C for grain; 50 °C with agitation for beer), the volatile compounds were extracted from the headspace for 40 min and then desorbed in the GC inlet for 5 min. All GC and MS parameters were set following previously established conditions [19].

#### 2.3.4. Determination of Antioxidant Indicators

Ground grain (1 g) was extracted with 5 mL of 80% ethanol via shaking and centrifugation, while filtered wort and beer were analyzed directly. The resulting samples were used to determine the TPC and antioxidant activities, including DPPH and ABTS radical scavenging capacities and ferric reducing antioxidant power (FRAP), following established methodologies [20].

### 2.4. Sensory Analysis

Twelve trained panelists (6 males and 6 females, aged 20–25 years) participated in the sensory evaluation, with training conducted with reference to our previous literature [10]. Wort for malt sensory evaluation was prepared according to the ASBC Hot Steep Method [21] by mixing 50 g of malt powder with 450 mL of water at 65 °C. After shaking for 20 s, the mixture was held for 15 min at the same temperature, stirred, and filtered to obtain the wort. Sensory evaluation for wort included three taste parameters (sweet, acid, and bitter) and six aroma parameters (burnt, nutty, caramel, coffee, smoky, and malty). For beer, the assessed attributes covered visual (foam, transparency, and color), taste (sweet, acid, bitter, alcohol, and carbonation), and aroma (malty, alcohol, hoppy, fruity, grassy, roasted, sulfury, and stale) dimensions. All wort samples were cooled to room temperature and served in 10 mL transparent plastic cups, whereas beer samples stored at 7 °C were poured into 20 mL cups immediately before evaluation. The intensity of each attribute was rated on a 9-point category scale, where 0 indicated “not perceptible,” 5 represented “medium strength,” and 9 denoted “extremely strong.”

### 2.5. Statistics

All experiments were performed with three biological replicates and three technical replicates per biological replicate. Chemical indices were compared between groups using an independent samples *t*-test, with statistical significance set at *p* < 0.05. Sensory data were analyzed using the Friedman test, followed by Bonferroni-corrected paired comparisons when significant (*p* < 0.05).

## 3. Results and Discussion

### 3.1. Effect of Explosive Puffing on Key Physicochemical Properties of Malt

**Figure 2:**
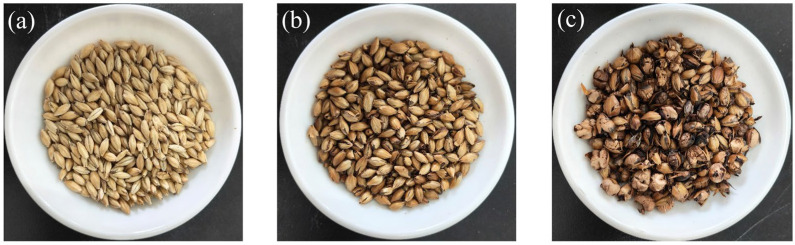
(**a**) Base malt. (**b**) Explosive puffed malt prepared at 0.8 MPa. (**c**) Explosive puffed malt prepared at 1.0 MPa.

As shown in Figure 2, at a pressure of 1.0 MPa, the EM exhibited marked expansion, whereas at 0.8 MPa, its volume remained comparable to that of BM, which could be attributed to insufficient pressure. However, the BM treated at 1.0 MPa showed evident charring and structural disruption, while that treated at 0.8 MPa displayed no significant charring and preserved its original morphology. Therefore, 0.8 MPa was selected as the processing condition for subsequent investigations. Based on the Light Industry Standard of the People’s Republic of China (QB/T 1686-2008 Barley Malt) [22], which stipulates moisture content, color, and extract as the key physicochemical indicators for specialty malts, these parameters were selected for analysis (Table 1). Following puffing, EM showed a significant reduction in moisture content, driven by the vaporization and expulsion of water under high temperature and pressure [23]. Meanwhile, grain color showed a substantial enhancement, due primarily to non-enzymatic browning during explosive puffing [24], though this color depth is lighter than that of 8 min microwave-treated barley malt [10]. Furthermore, a significant decrease in extract content was observed. This phenomenon might be attributed to the volatilization of volatile products generated by intense non-enzymatic browning under the high-temperature conditions of explosive puffing.

### 3.2. Effect of Explosive Puffing on Volatile Profile of Malt

The explosive puffing process induced profound alterations in the volatile composition of grain, as detailed in Table 2. While the BM was characterized by a simpler profile dominated by aldehydes and alcohols, the EM exhibited a more complex aroma profile rich in pyrazines, furans, and other compounds. The total concentration of volatile compounds was also substantially higher in the EM compared to the BM.

Pyrazines are nitrogen heterocyclic aromatic compounds featuring diverse substituents. During thermal processing, they are generated through the condensation of aminoketones, which are co-products of Strecker degradation [25]. In specialty malt, pyrazines are considered key odorants and have been widely recognized in numerous studies for their significant contribution to flavor [26]. Comparing samples before and after explosive puffing, pyrazines were the most significantly impacted group. No pyrazines were detected in the BM sample, whereas a total of 8 pyrazine compounds were identified in the EM sample. Key compounds such as 2-methylpyrazine, 2,6-dimethylpyrazine, and 2-ethylpyrazine are well-known for their strong roasted and nutty aromas. In contrast, microwave-treated malt generates pyrazines that peak at 4 min of treatment and decrease with prolonged heating, while explosive puffing produces a higher total concentration of pyrazines in one step without subsequent degradation or volatilization [10]. Similarly, pyrroles, another kind of nitrogen heterocyclic compounds, represented by 2-acetylpyrrole (popcorn) and 1h-pyrrole-2-formaldehyde (roasted bread), were only found in the EM sample, further enhancing the complex roasted character. Furans, a class of aroma-active oxygen heterocyclic compounds, comprised the most abundant group of volatiles in EM. A dramatic and significant increase was observed for furfural, which is a key product of sugar dehydration. Other important furan compounds generated during puffing included 5-methylfurfural and 2-acetylfuran. These compounds impart sweet, caramel, and bready notes, and their concentrations in EM are generally higher than those in microwave-treated malt, contributing to a more intense caramel aroma [10].

Beyond the abundant generation of heterocyclic compounds, other volatile classes also underwent significant changes. The profile of aldehydes shifted. Hexanal, which gives a “grass” odor and was the dominant aldehyde in BM, was completely absent in the EM sample. This suggests that the explosive puffing process degraded this compound, which is associated with lipid oxidation and fresh, green notes. Ketones also showed a marked increase in EM, driven by the thermal degradation of proteins and fatty acids, deamination of amino acids, and decarboxylation of carboxylic acids during heating, among which 3,5-dihydroxy-6-methyl-2,3-dihydropyran-4-one being a notable contributor to the caramel flavor.

### 3.3. Effect of Explosive Puffing on Sensory Attributes of Malt

Table 3 presents the sensory evaluation scores of the BM and EM. The sweet, a dominant characteristic of the BM, decreased significantly in the EM. This reduction was likely due the intense non-enzymatic browning reactions during explosive puffing. The bitter and roasted flavor compounds generated both mask the sweet taste and are responsible for the elevated bitter score. An increase in the acid score was observed in the EM, a result in line with prior studies which document that dark malts generally have a lower pH than pale malts [27].

The most dramatic transformation occurred in the aroma profile. The characteristic malty aroma, which was dominant in the BM, was significantly subdued in the EM, as it was overshadowed by a suite of newly formed aroma compounds. All aroma attributes associated with roasting exhibited significant increases. The most pronounced change was observed in caramel aroma, making it the strongest aroma note in the EM. This finding is supported by the volatile analysis, which showed a high concentration of furans like 5-methylfurfural (almond, caramel), and ketones like 3,5-dihydroxy-6-methyl-2,3-dihydropyran-4-one (caramel). Similarly, the coffee and nutty score increased. These sensory results are explained by the formation of pyrazines in the EM sample, such as 2-methylpyrazine and 2,6-dimethylpyrazine, which are renowned for their nutty and roasted coffee-like aromas. The development of smoky and burnt notes indicates the intense thermal load of the explosive puffing process.

### 3.4. Effect of Explosive Puffed Malt Addition on Physicochemical Properties of Wort and Beer

To preliminarily evaluate the applicability of EM, we employed it in the preparation of wort and beer. Given the conventional threshold of 30% for specialty malt in brewing research [28], a moderate proportion of 15% was therefore employed (Table 4). The 15% EM batch exhibited a significant decrease in pH in both the wort and beer, a finding that aligns with the acidifying effect of dark malt documented in numerous studies [29,30]. The intense color contributed by the EM was retained from the wort stage through to the final beer. The concentrations of FAN and reducing sugars showed no significant differences between the two worts. This is an intriguing finding. It is hypothesized that the stability of the measured FAN is attributable to a compensatory effect. While the Maillard reaction during explosive puffing consumes a portion of the free amino acids and small peptides, the intense thermo-mechanical stress simultaneously induces the denaturation and partial hydrolysis of larger, un-degraded proteins in the malt [23]. These complex proteins, which are not fully broken down during the mashing process, are degraded into smaller peptides and amino acids that are detectable by the ninhydrin method used for FAN analysis. Consequently, the consumption of FAN via the Maillard pathway is counterbalanced by the generation of FAN from protein breakdown. Similarly, the lack of a significant decrease in reducing sugar concentration is hypothetically explained by an equilibrium between consumption and generation. The Maillard reaction consumes reducing sugars. However, the explosive puffing causes the thermal degradation of non-starch polysaccharides [23]. These carbohydrates, not fully degraded under mashing conditions, are broken down under high temperature and pressure into their monomeric and oligomeric subunits, which are quantified as reducing sugars. Thus, the loss of sugars to color and flavor formation is hypothesized to be offset by the gain in sugars from the depolymerization of non-starch polysaccharides. Alcohol content showed no significant variation. This consistency could be attributed to the stable reducing sugar levels in wort, which provided sufficient fermentable substrates for yeast metabolism to synthesize alcohol. A consistent hopping rate was maintained across all batches, resulting in comparable bitterness intensities in the final beers. The significant turbidity increase in the 15% EM beer implicates the introduction of haze-forming compounds from the EM, such as proteins, polyphenols, or high-molecular-weight melanoidins [31].

### 3.5. Effect of Explosive Puffed Malt Addition on Volatile Profile of Beer

The volatile compounds in two types of beer samples were analyzed, as presented in Table 5. Higher alcohols significantly contribute to the sensory profile of beer. The 0% EM and 15% EM beers contained similar levels of higher alcohols. Furfuryl alcohol, a compound with a strong caramel-like odor, was present at a six-fold higher concentration in the 15% EM beer. In a study analyzing beers brewed with varying proportions of Caraamber^®^ malt (a type of dark malt), the concentration of furfuryl alcohol progressively increased with the usage level of the dark malt [32]. Esters are responsible for the characteristic fruity and floral notes in beer. The elevated ester content in the 15% EM beer was mainly attributable to an increase in banana-like isoamyl acetate, stemming from a higher concentration of its precursor, isoamyl alcohol. High acidity in beer adversely affects its flavor, especially when exceeding the sensory threshold. While the 15% EM beer contained a higher concentration of octanoic acid than the 0% EM variant, its level remained below the threshold of 13 mg/L. Furthermore, 2-acetylfuran, a compound implicated in the character of various specialty malts [33], was unique to the 15% EM beer, indicating that certain thermal degradation products from the EM could carry over into the final beer.

### 3.6. Effect of Explosive Puffed Malt Addition on Sensory Characteristics of Beer

Table 6 summarizes the sensory evaluation results for the two beer samples, with attributes classified into visual, taste, and aroma categories. Significant differences were observed in all visual attributes. The color of the 15% EM beer was rated significantly higher than the 0% EM beer, which aligns with the increase in wort and beer color. The transparency of the 15% EM beer was significantly lower, corroborating the increased turbidity detected in the physicochemical analysis. Furthermore, the foam score was significantly improved in the 15% EM beer. Preliminary evidence suggests the presence of foam-stabilizing components in roasted barley and black malt extract [34]. In terms of taste, the 15% EM beer was perceived as significantly more acidic, a finding consistent with its lower pH. The carbonation was also rated significantly higher, which could be related to the improved foam stability. The aroma profile exhibited a distinct evolution, marked by a decline in the malty and grassy scores of control beer and a concurrent rise in the roasted notes characteristic of dark beers. The fruity score was also significantly enhanced, which could be attributed to the higher concentration of esters, as showed by the volatile compound analysis.

### 3.7. Effect of Explosive Puffing on Antioxidant Properties of Malt, Wort and Beer

The antioxidant properties of cereal, wort, and beer are presented in Table 7. The EM demonstrated a remarkable increase in all antioxidant metrics compared to the BM. By promoting internal macromolecular breakage, explosive puffing facilitates the release of polyphenols and other chemicals, which in turn yields a corresponding increase in antioxidant activity. This outcome corroborates earlier work describing the effects of explosive puffing on coffee beans [35]. Additionally, the various compounds generated during non-enzyme browning under high temperatures serve as another source of its antioxidant activity. The elevated antioxidant potential of the EM was successfully transferred to the wort and the final beer, despite the EM constituting only 15% of the grist. The observed decline in absolute values from malt to beer is expected due to dilution, precipitation, and adsorption of compounds during mashing, boiling, and fermentation.

### 3.8. Limitations and Future Perspectives

Despite the promising results, this study has several limitations. The beer brewed with 15% puffed malt exhibited significantly higher turbidity compared to the control, which may affect consumer acceptance and requires further clarification. Moreover, while explosive puffing proved effective at the laboratory scale, data on its industrial scalability are lacking. Future research should focus on optimizing processing parameters to minimize haze formation, as well as conducting pilot-scale trials to validate the technology for commercial application.

## 4. Conclusions

In summary, this study demonstrates that explosive puffing is an innovative approach for producing specialty malt, which efficiently converts base malt into a product with a distinct caramel-roasted aroma and enhanced antioxidant capacity, and can be feasibly incorporated into brewing at a 15% grist ratio. This application significantly enriches the color and flavor of wort and beer, and maintains levels of free amino nitrogen and reducing sugars, offering a promising alternative to conventional roasting methods.

## Figures and Tables

**Table 1:** Grain indicators.

Indicators	BM	EM
Moisture %	8.71 ± 0.41 *	4.94 ± 0.14
Color EBC	4.00 ± 0.03	183.15 ± 3.56 *
Extract %	79.59 ± 0.48 *	70.39 ± 1.24

* denotes significant differences between the two groups in the same row (*p* < 0.05).

**Table 2:** Volatile substances of grain.

Classification	Compound	RT /min ^a^	Content (mg/kg)		Prob ^b^ (%)	RI ^c^	Description ^d^	Identification
			BM	EM				
Pyrazines								
1	Pyrazine	8.06	-	0.035 ± 0.004	58.1	1215/1232	Roasted hazelnut	RI, MS
2	2-Methylpyrazine	10.17	-	1.689 ± 0.095	85.1	1267/1288	Baking, cocoa	RI, MS
3	2,5-Dimethylpyrazine	12.65	-	0.641 ± 0.028	78.7	1321/1348	Peanuts, chocolate	RI, MS
4	2,6-Dimethylpyrazine	12.92	-	0.977 ± 0.052	74.4	1327/1354	Roasted nuts, cocoa	RI, MS
5	2-Ethylpyrazine	13.09	-	1.043 ± 0.038	68.1	1330/1359	Peanut sauce	RI, MS
6	2,3-dimethylpyrazin	13.71	-	0.251 ± 0.022	84.9	1342/1371	Nuts, cocoa	RI, MS
7	2-Ethyl-6-methylpyrazine	15.68	-	0.699 ± 0.025	71.2	1381/1408	Roasted, coffee	RI, MS
8	2,3,5-Trimethylpyrazine	16.67	-	0.553 ± 0.089	85.8	1401/1407	Nuts, foxtail	RI, MS
10	Acetylpyrazine	24.37	-	0.110 ± 0.009	81	1604/1621	Baking	RI, MS
	Total		0	5.998				
Pyrroles								
1	2-Acetylpyrrole	29.25	-	0.310 ± 0.045	73.2	1956/2006	Popcorn	RI, MS
2	1H-pyrrole-2-formaldehyde	29.83	-	0.164 ± 0.019	92.1	2007/2032	Roasted bread	RI, MS
	Total		0	0.474				
Furans								
1	2-Pentylfuran	9.1	-	0.069 ± 0.012	60.8	1244/1230	Butter, green beans	RI, MS
2	Furfural	19.31	0.045 ± 0.001	6.766 ± 0.614 *	78	1453/1460	Bread, almond	RI, MS
3	2-Acetylfuran	21.14	-	1.546 ± 0.009	80.3	1490/1500	Nut, caramel	RI, MS
4	2-Butyl furan	21.77	-	0.049 ± 0.005	59.7	1504/1112	Fruit wine	RI, MS
5	5-Methylfurfural	23.19	-	2.278 ± 0.112	95.4	1553/1570	Almond, caramel	RI, MS
6	2 (5H)-furanone	26.41	-	0.097 ± 0.004	92	1720/1703	Buttery	RI, MS
7	5-Hydroxymethyl furfural	36.1	-	0.125 ± 0.049	85	2497/2485	Cardboard	RI, MS
	Total		0.045	10.93				
Aldehyde								
1	Hexanal	4.98	1.036 ± 0.012	-	60.2	1111/1089	Grass	RI, MS
2	Benzaldehyde	21.64	-	0.114 ± 0.010	56.7	1499/1503	Almond, nut	RI, MS
3	Phenylacetaldehyde	24.6	-	0.092 ± 0.071	72.8	1602/1623	Honey, cocoa	RI, MS
	Total		1.036	0.206				
Ketones								
1	Hydroxyacetone	11.34	-	0.301 ± 0.05	81.1	1293/1295	Truffle, nut	RI, MS
2	4-Cyclopentene-1,3-dione	23.35	-	0.369 ± 0.023	93.6	1558/1535	Smoky	RI, MS
3	3,5-Dihydroxy-6-methyl-2,3-dihydropyran-4-one	32.89	-	0.830 ± 0.162	96.3	2262/2295	Caramel	RI, MS
	Total		0	1.5				RI, MS
Alcohols								
1	Isoamyl alcohol	8.5	0.238 ± 0.014	-	67.2	1224/1200	Malt, charred	RI, MS
2	1-Pentanol	10.11	0.120 ± 0.006	-	53.4	1264/1260	Bitterness	RI, MS
3	Furaneol	30.03	-	0.403 ± 0.035	63.6	2024/2007	Caramel	RI, MS
	Total		0.358	0.403				
Acids								
1	Acetic acid	18.23	0.074 ± 0.010	-	81.8	1431/1465	Sour	RI, MS
2	Butyric acid	24.73	-	0.100 ± 0.009	61.9	1625/1630	Cheese	RI, MS
3	Hexanoic acid	28.21	0.051 ± 0.001	-	52.9	1866/1833	Sour	RI, MS
	Total		0.125	0.1				
Esters								RI, MS
1	Furfuryl acetate	22.42	-	0.256 ± 0.014	88	1526/1552	Fruity, spicy	RI, MS
2	γ-Butyrolactone	24.21	-	0.098 ± 0.074	72.9	1588/1665	Buttery	RI, MS
	Total		0	0.354				
Phenols								
1	4-Vinyl-2-methoxyphenol	32.06	-	0.075 ± 0.016	54.2	2194/2200	Clove, curry	RI, MS
	Total		0	0.075				

* denotes significant differences between the two groups in the same row (*p* < 0.05), “-” represents not detected. ^a^ Retention time on a Thermo-TG-Wax MS capillary column; ^b^ the compound was identified by prob value over 50% and matching with the RI; ^c^ RI relative to C_7_–C_30_ n-alkanes was calculated on DB-WAX capillary column, the retention index value in the literature was searched from https://webbook.nist.gov/chemistry/name-ser/ (accessed on 25 June 2025); ^d^ Aroma description was come from https://www.flavornet.org/flavornet.html (accessed on 25 June 2025).

**Table 3:** Sensory evaluation of malt.

Attribute	BM	EM
**Taste**		
Sweet	7.92 ± 0.64 *	4.25 ± 0.83
Acid	1.08 ± 0.86	3.58 ± 1.06 *
Bitter	1.42 ± 0.95	5.42 ± 1.19*
**Aroma**		
Burnt	1.21 ± 0.75	3.46 ± 0.69 *
Nutty	1.25 ± 0.43	3.92 ± 0.86 *
Caramel	2.00 ± 0.91	6.54 ± 1.22 *
Coffee	1.08 ± 0.76	5.13 ± 1.32 *
Smoky	0.83 ± 0.55	3.79 ± 0.90 *
Malty	7.08 ± 0.95 *	4.42 ± 1.75

* denotes significant differences between the two groups in the same row (*p* < 0.05).

**Table 4:** Physicochemical indicators of wort and beer.

	Indicators	0% EM	15% EM
Wort	pH	5.86 ± 0.08 *	5.59 ± 0.05
	Color EBC	9.71 ± 0.03	48.71 ± 1.13 *
	FAN mg L^−1^	222.92 ± 1.70	220.99 ± 2.20
	Reducing sugar g 100 mL^−1^	8.90 ± 0.05	8.87 ± 0.20
Beer	Alcohol %vol	3.66 ± 0.05	3.71 ± 0.05
	Fermentation degree %	57.95 ± 0.09 *	56.61 ± 0.49
	pH	4.29 ± 0.02 *	4.20 ± 0.02
	Color EBC	8.59 ± 0.14	48.45 ± 0.52 *
	Bitterness IBU	13.40 ± 0.43	13.92 ± 0.43
	Turbidity EBC	17.84 ± 0.52	21.03 ± 0.81 *

* denotes significant differences between the two groups in the same row (*p* < 0.05).

**Table 5:** Volatile substances of beer.

Classification	Compound	RT/min ^a^	Content (mg/L)		Prob ^b^ (%)	RI ^c^	Description ^d^	Identification
			0% EM	15% EM				
Alcohols								
1	Isoamyl alcohol	9.35	19.87 ± 0.96	22.31 ± 1.11 *	64.9	1245/1236	Floral, fruity	RI, MS
2	1-Nonen-3-ol	21	-	0.19 ± 0.01	51.8	1487/1114	Mushroom	MS
3	1-Heptanol	21.2	0.14 ± 0.00	-	52.6	1491/1487	Oil, spicy	RI, MS
4	Linalool	23.8	0.14 ± 0.00	0.19 ± 0.00 *	55	1582/1576	Floral, fruity	RI, MS
5	1-Octanol	24.1	0.67 ± 0.02	0.72 ± 0.02	68.2	1591/1586	Oil, citrus	RI, MS
6	Furfuryl alcohol	25.9	0.09 ± 0.00	0.54 ± 0.03 *	75.7	1690/1661	Caramel	RI, MS
7	5-Methylfurfuryl alcohol	26.8	-	0.05 ± 0.01	90.3	1754/1729	Caramel	RI, MS
8	1-Decanol	27.4	0.17 ± 0.01	0.18 ± 0.03	50.2	1798/1639	Floral, fruity	RI, MS
9	2-Phenylethanol	29.2	22.81 ± 1.06 *	20.44 ± 0.31	83.8	1952/1898	Rose, flower	RI, MS
	Total		43.89	44.72				
Esters								
1	Isoamyl acetate	6.5	59.64 ± 1.67	72.27 ± 8.81 *	87.2	1165/1127	Banana	RI, MS
2	Ethyl caproate	10.3	23.20 ± 0.75 *	19.18 ± 1.36	83.2	1268/1236	Wine, fruit	RI, MS
3	Ethyl octanoate	20.1	8.50 ± 1.45	6.35 ± 1.35	84.8	1468/1424	Floral, fruity	RI, MS
4	Furfuryl acetate	23.4	-	0.15 ± 0.00	90.2	1567/1531	Fruity	RI, MS
5	Ethyl decanoate	25.5	0.26 ± 0.05	0.21 ± 0.03	72.3	1669/1638	Fruity	RI, MS
6	Ethyl 9-decenoate	26.3	0.06 ± 0.00	-	60.8	1716/1668	Fruit	RI, MS
7	Phenethyl acetate	28.1	7.67 ± 0.27	8.57 ± 0.23 *	57.7	1854/1821	Rose, fruit	RI, MS
8	Ethyl 3-phenylpropionate	28.9	-	0.06 ± 0.00	50.5	1926/1872	Flower	RI, MS
	Total		99.33	106.73				
Acids								
1	Acetic acid	20.8	-	0.45 ± 0.18	81.9	1483/1451	Sour	RI, MS
2	Butyric acid	25.4	0.02 ± 0.00	0.04 ± 0.00 *	71.8	1661/1642	Cheese	RI, MS
3	Hexanoic acid	28.4	1.24 ± 0.11	1.62 ± 0.33	82.5	1882/1846	Fruit, cheese	RI, MS
4	Octanoic acid	31	3.42 ± 0.25	4.77 ± 0.35 *	90.8	2104/2092	Sour	RI, MS
5	Decanoic acid	33.8	0.12 ± 0.01	0.22 ± 0.02 *	56.3	2272/2266	Fatty	RI, MS
	Total		4.8	7.1				
Phenols								
1	4-Vinyl-2-methoxyphenol	32.7	-	0.05 ± 0.01	59.5	2249/2214	Clove, smoked	RI, MS
2	2,4-Di-tert-butylphenol	34.2	0.26 ± 0.01 *	0.19 ± 0.01	56.8	2358/2321	Phenolic	RI, MS
	Total		0.26	0.24				
Others								
1	2-Acetylfuran	22.4	-	0.42 ± 0.00	66.7	1529/1534	Balsam	RI, MS
2	Phenylacetaldehyde	25.4	0.04 ± 0.01	-	66.4	1664/1674	Hawthorn, honey	RI, MS
	Total		0.04	0.42				

* denotes significant differences between the two groups in the same row (*p* < 0.05), “-” represents not detected. ^a^ Retention time on a Thermo-TG-Wax MS capillary column; ^b^ the compound was identified by prob value over 50% and matching with the RI; ^c^ RI relative to C_7_–C_30_ n-alkanes was calculated on DB-WAX capillary column, the retention index value in the literature was searched from https://webbook.nist.gov/chemistry/name-ser/ (accessed on 25 June 2025); ^d^ Aroma description was come from https://www.flavornet.org/flavornet.html (accessed on 25 June 2025).

**Table 6:** Sensory evaluation of beer.

Attribute	0% EM	15% EM
**Visual**		
Foam	5.17 ± 0.59	6.33 ± 0.62 *
Transparency	6.79 ± 0.75 *	4.75 ± 1.01
Color	3.92 ± 0.76	6.67 ± 0.75 *
**Taste**		
Sweet	1.75 ± 0.69	2.08 ± 0.61
Acid	4.08 ± 0.73	5.33 ± 0.72 *
Bitter	5.92 ± 0.73	6.67 ± 0.82
Alcohol	5.25 ± 0.60	5.67 ± 0.62
Carbonation	5.58 ± 0.49	6.42 ± 0.76 *
**Aroma**		
Malty	6.83 ± 0.69 *	3.33 ± 0.62
Alcoholic	6.06 ± 0.50	6.08 ± 0.76
Hoppy	4.67 ± 0.94	4.33 ± 0.47
Fruity	5.67 ± 0.85	6.92 ± 0.76 *
Grassy	3.67 ± 0.85 *	1.67 ± 0.75
Roasted	2.08 ± 0.86	7.67 ± 0.75 *
Sulfury	1.58 ± 0.49	1.5 ± 0.96
Stale	1.85 ± 0.64	1.82 ± 0.55

* denotes significant differences between the two groups in the same row (*p* < 0.05).

**Table 7:** Assessment of antioxidant properties.

	BM	EM
**Grain**		
TPC mg GAE L^−1^	487.59 ± 4.43	1450.71 ± 8.86 *
FRAP mmol TE L^−1^	2.87 ± 0.06	11.62 ± 0.23 *
ABTS mmol TE L^−1^	3.62 ± 0.01	10.55 ± 0.33 *
DPPH mmol TE L^−1^	0.78 ± 0.03	2.64 ± 0.05 *
**Wort**		
TPC mg GAE L^−1^	476.24 ± 11.72	609.57 ± 6.39 *
FRAP mmol TE L^−1^	1.68 ± 0.04	2.78 ± 0.13 *
ABTS mmol TE L^−1^	4.49 ± 0.10	5.14 ± 0.09 *
DPPH mmol TE L^−1^	0.45 ± 0.02	0.60 ± 0.01 *
**Beer**		
TPC mg GAE L^−1^	361.35 ± 6.14	516.67 ± 3.25 *
FRAP mmol TE L^−1^	1.71 ± 0.04	2.54 ± 0.06 *
ABTS mmol TE L^−1^	2.85 ± 0.10	3.65 ± 0.10 *
DPPH mmol TE L^−1^	0.37 ± 0.02	0.64 ± 0.01 *

* Denotes significant differences between the two groups in the same row (*p* < 0.05). EM was incorporated at 15% addition levels in wort and beer. Values are means of triplicate ± standard deviation.

## Data Availability

The original contributions presented in this study are included in the article. Further inquiries can be directed to the corresponding author.
